# Ionic Mobility in Ion-Exchange Membranes

**DOI:** 10.3390/membranes11030198

**Published:** 2021-03-11

**Authors:** Irina A. Stenina, Andrey B. Yaroslavtsev

**Affiliations:** Kurnakov Institute of General and Inorganic Chemistry of the Russian Academy of Sciences, Leninsky pr. 31, 119991 Moscow, Russia; stenina@igic.ras.ru

**Keywords:** ion-exchange membrane, hybrid membrane, mobility, NMR spectroscopy, diffusion, ionic conductivity, proton transfer, hydration

## Abstract

Membrane technologies are widely demanded in a number of modern industries. Ion-exchange membranes are one of the most widespread and demanded types of membranes. Their main task is the selective transfer of certain ions and prevention of transfer of other ions or molecules, and the most important characteristics are ionic conductivity and selectivity of transfer processes. Both parameters are determined by ionic and molecular mobility in membranes. To study this mobility, the main techniques used are nuclear magnetic resonance and impedance spectroscopy. In this comprehensive review, mechanisms of transfer processes in various ion-exchange membranes, including homogeneous, heterogeneous, and hybrid ones, are discussed. Correlations of structures of ion-exchange membranes and their hydration with ion transport mechanisms are also reviewed. The features of proton transfer, which plays a decisive role in the membrane used in fuel cells and electrolyzers, are highlighted. These devices largely determine development of hydrogen energy in the modern world. The features of ion transfer in heterogeneous and hybrid membranes with inorganic nanoparticles are also discussed.

## 1. Introduction

Membrane technologies are widely demanded in a number of modern industries. They are used for gas separation [1,2,3], water purification [4,5,6,7], purification of pharmaceutical drugs and biological fluids [8]. The number of applications of membrane technologies in the chemical and petrochemical industries [9,10,11,12,13,14,15], modern energy [16,17,18,19,20,21,22,23], and sensorics [24,25,26,27] has significantly increased. Ion-exchange membranes are one of the most widespread and demanded membrane types [5,28]. Their main task is the selective transfer of certain ions and prevention of transfer of other ions or molecules. For example, separation of ions with different charges is of most interest in electrodialysis [29,30]. At the same time, the concept of zero liquid discharge becomes an increasingly acute issue. This is dictated not only by environmental requirements—the problem of extracting valuable components from wastewater and processed products has attracted much attention [31,32,33,34]. In this case, the issue of separation of mono- and divalent ions is becoming more and more important [35,36,37,38,39,40,41,42]. For example, membrane separation of lithium and magnesium or cobalt ions [43,44,45,46,47] and the removal of magnesium/calcium [48,49,50,51] have attracted increasing attention. On the contrary, membranes in fuel cells are usually in single ionic form (H^+^ or OH^−^-form) and contact only with humidified gases or methanol. During fuel cell operation, only the same ions (protons or OH^−^-ions) are generated [52,53,54]. Nevertheless, even in this case, it is necessary to limit gas diffusion through the membrane as much as possible, which determines the so-called fuel crossover—the undesired passage of fuel, not accompanied by energy production [55,56,57]. The ratio of fluxes of desired and undesired components determines the value of selectivity, which is one of the most important membrane operating parameters [37,58]. Selectivity is determined by the ratio of the mobility of different ions or molecules in the membrane. At the same time, the rate of target component transfer, determined by its mobility, is a no less important parameter [59,60,61]. It is this parameter that determines the performance of different electromembrane processes [62,63].

This shows the importance of investigation of ionic and molecular mobility in ion-exchange membranes [60,64]. Impedance spectroscopy is undoubtedly the main method for studying ionic mobility in solids [65,66,67,68], but it usually provides information on the total conductivity. Blocking electrodes can be used to separate the contributions of different ions [69], although this significantly complicates the measurement procedure. It is not always a simple task to separate the contributions of the bulk and grain-boundary conductivity, as well as that of the electrolyte/electrode interface [70,71]. Moreover, impedance spectroscopy provides information only on the translational mobility of ions.

Ionic mobility can also be investigated by nuclear magnetic resonance (NMR) spectroscopy [72,73]. This method allows studying of mobility of various nuclei, which is a huge advantage. Moreover, NMR spectroscopy allows one to characterize the local environment of a particular nucleus and to characterize not only translational but also rotational mobility [74,75,76,77]. This makes it an indispensable tool for studying mechanisms of transfer processes, which can include different stages. Unlike impedance spectroscopy, NMR allows one to describe molecular mobility [78]. This provides an undeniable advantage, especially when studying transfer processes in ion-exchange membranes, in which the mobility of solvent molecules plays an important role [23,79,80]. Significant advantages are provided by the joint analysis of data obtained using impedance and NMR spectroscopy.

The purpose of this review is to consider the regularities and features of transfer processes in different ion-exchange membranes, including homogeneous, heterogeneous, and hybrid ones.

## 2. Ionic Transport in Solids and Membranes

We are used to considering solids as crystalline structures with an ideal order, in which all atoms and ions are fixed in their own positions, and only electrons can be mobile. According to the laws of thermodynamics, defects are always present in solids—vacancies (lattice sites that would be occupied) or atoms or ions in sites that would not contain anything (the so-called interstitials) [81,82]. Ions cannot move to another site in the ideal close-packed crystal. Such a jump should be associated with a high activation energy due to a sharp increase in repulsive forces with a decrease in the interionic distance. It is the defects that provide ion mobility (the movement of an ion to a vacant site leads to a charge transfer). For this, ions should cross the coordination polyhedron edge, which is characterized by the minimum size, the so-called “bottleneck”. Interionic distances in this position are significantly shorter, and the ion energy is much higher. The lifetime of ions in this state is negligible. Therefore, it is usually assumed that ion transfer in solids occurs via fast hopping between adjacent positions. The difference in energy between the bottleneck and the regular site determines the activation energy for ion migration (*E_m_*).

In the presence of an external electric field, ionic transfer in the crystal becomes ordered, and its ionic conductivity (*σ*) is determined by the equation:

*σ* = (*Cqa*^2^*ν*_0_*kT*) *exp*(−*E_m_/kT*), (1)

where *C* is the concentration of charge carriers with the charge *q*, a is the hopping length along the chosen direction, *ν*_0_ is the pre-exponential factor that determines the ion hopping frequency, *k* is the Boltzmann constant, and *T* is the absolute temperature (in Kelvin degrees). Expressing defect concentration in terms of the enthalpy (*ΔH_d_*) and entropy (*ΔS_d_*) of defect formation, Equation (1) can be rewritten in the form [82]:

*σ* = (*qa*^2^*ν*_0_/*kT*) *exp*(*ΔS*_*d*_/*pk*) *exp*[−(*Em* + *ΔH*_*d*_/*p*)/*kT*], (2)

where *p* is the number of ions formed during one act of defect formation (for ionic crystal *A^n+^X^m−^*, *p= n + m*). Since it is rather difficult to determine all the parameters of this expression, it is often replaced by the Frenkel (Equation (3)) or Arrhenius (Equation (4)) equations [82]:

*σT* = *Aexp*(−*E_σ_*/*kT*), (3)

*σ* = *σ*_0_*exp*(−*E*_*σ*_/*kT*), (4)

where *A* and *σ*_0_ are the pre-exponential factors and *E_σ_* is the activation energy of ionic conductivity. The last equation is used for rather narrow temperature ranges. The activation energy of conductivity thus includes the activation energy of ion migration (*E_m_*) and an additional contribution of the enthalpy of defect formation (*ΔH_d_/p*).

The features of ionic transfer in ion-exchange membranes can be shown using the example of homogeneous perfluorosulfonic acid membranes of the Nafion type, the structure of which is usually described by the Gierke model [83,84]. Nafion is a copolymer of tetrafluoroethylene and perfluorinated sulfonated vinyl ether and presents a perfluorinated polymer chain with side chains containing the –SO_3_H terminal groups [85]. In contrast to a hydrophobic matrix, functional groups are hydrophilic and form clusters as a result of self-organization. Due to the absorption of water from the contacting solution or the atmosphere, the clusters swell, forming a system of pores and channels in the membrane. This network of pores and channels is filled with water, and pore walls are lined with highly acidic –SO_3_H groups. As a result of their dissociation, the pore walls acquire a negative charge, while protons in aqueous solution provide membrane conductivity [86]. 

According to the small-angle X-ray scattering data, the membrane pores in the swollen state have sizes of 4–5 nm, and the sizes of channels connecting them are 1–2 nm [87,88]. The proton-acceptor ability of –SO_3_^−^ groups is significantly lower than that of water molecules, due to which SO_3_H groups dissociate, forming hydrated proton (H^+^(H_2_O)_n_). This is confirmed by the NMR spectroscopy data [89]. At the same time, the number of water molecules per functional group (the membrane hydration degree) is usually 10–18 for Nafion membranes in a swollen state, which is much more than necessary for the formation of stable proton hydrates with a proton hydration degree of 1–4. It can be assumed that an ice-like structure of water with H^+^(H_2_O)_n_ ions as defects is formed in the membrane pores. The number of such defects remains constant and does not depend on temperature. Therefore, the activation energy of conductivity is determined by the activation energy of ion migration only and decreases with increasing membrane water uptake.

As mentioned above, membrane transport properties can be studied using both conductivity measurements and NMR spectroscopy. If impedance spectroscopy is used for studying ion transport across a sample, NMR spectroscopy provides specific information about local dynamics of ions. Both the pulsed field gradient NMR (PFG-NMR) method and different spin-relaxation techniques can be used for investigation of diffusion coefficients [72,76,79]. The relationship between the conductivity (*σ_i_*) and the diffusion coefficient D_i_ for any given ions *i* is established by the Nernst–Einstein equation [90,91,92]:

*D_i_* = *RT*/*z_i_^2^F^2^σ_i_*, (5)

where *z_i_* is the charge of ion *i*, *F* is the Faraday constant, and *R* is the gas constant. This equation is used to calculate the ionic diffusion coefficients from experimental determinations of conductivity or vice versa.

## 3. Hydration and Mobility of Cations in Membranes

The water uptake of ion-exchange membranes determines all their practically important properties and, first of all, transfer processes. The membrane water uptake is determined by a number of parameters, among which the most important are the relative humidity, the nature of the counterions and fixed ions, and the hydrophilicity and rigidity of the polymer matrix of the membrane [93,94]. This review is focused on cation-exchange membranes, in which cations are most actively involved in the membrane hydration. The membrane water uptake is primarily determined by the polarizing power of a cation. In the series of singly charged ions, the proton is the most intensively hydrated. For alkali metal cations, the degree of hydration decreases with an increase in the cation radius from lithium to cesium. The NMR data [89,95] provide important information on these processes. At a low membrane hydration degree (<4–6 water molecules per functional group), the ^7^Li and ^23^Na chemical shifts change rather quickly due to formation of contact ion pairs between membrane functional groups and metal ions, since there are not enough water molecules to saturate their coordination spheres [96,97]. At higher hydration degrees, the environment of cations in membranes becomes similar to that for aqueous solutions of their salts, and the NMR chemical shifts approach these for aqueous solutions. The energy of interactions of large cesium cations with water molecules is insufficient to destroy the network of hydrogen bonds of water molecules, and the dependence of the ^133^Cs chemical shift values on hydration degree is not pronounced [96,98]. This conclusion correlates well with dependences of ^1^H chemical shifts on hydration degree [99,100].

**Figure 1:**
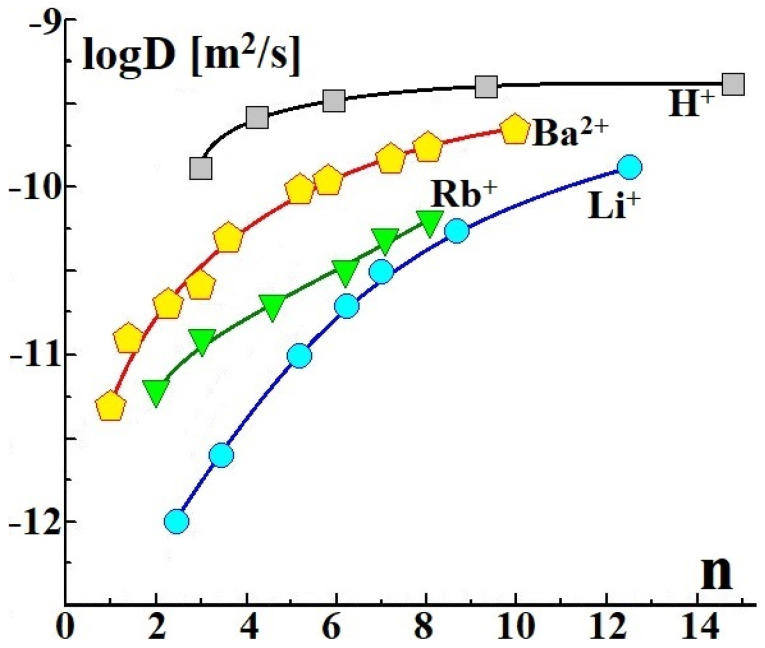
Dependences of diffusion coefficients of proton-containing groups (water self-diffusion coefficients) on hydration degree (number of water molecules per functional group) for perfluorinated sulfonic acid MF-4SK membranes in different ionic forms [101].

Using ^1^H pulsed field gradient NMR, it was shown that at the same hydration degrees of membranes, the water diffusion coefficients decreased in the sequence H^+^ > Ba^2+^ > Cs^+^ > Na^+^ > Li^+^ (Figure 1) [99,100,101]. In NMR studies, it is usually called the water self-diffusion coefficient. For alkali metal ions, this is due to the stronger binding of water with a decrease in the cation radius. However, at the same relative humidity, the membrane water uptake increases in the same sequence, which, on the contrary, leads to an increase in water mobility. Obviously, barium ions bind water molecules even more strongly. 

It thus seems unreasonable that at close degrees of hydration, water mobility is even higher in the Ba^2+^-forms of membranes than that in the Cs^+^-forms (Figure 1). However, barium ions are doubly charged, and the number of cations per water molecule in membranes is half the number of alkali metal cations. Thus, the amount of weakly bound water is larger, which leads to a higher mobility of water molecules in the Ba^2+^-forms of membranes. We will discuss the reasons for the higher water mobility in the H^+^-forms of membranes a little later.

**Figure 2:**
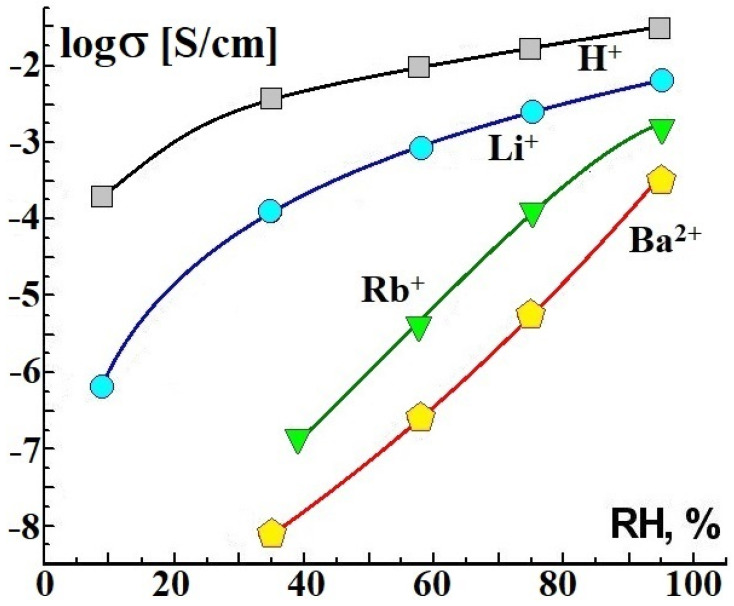
Dependences of ionic conductivity on relative humidity for perfluorinated sulfonic acid MF-4SK membranes in different ionic forms [101].

It appears logical to conclude that ionic conductivity of membranes in different ionic forms should correlate with the mobility of water molecules. Even more surprising is that for membranes kept at the same relative humidity, the dependence of the conductivity on the cation radius is strictly opposite (Figure 2) [101,102,103,104]. The water absorption of membranes at equivalent relative humidity is directly related to the polarizing power of an ion and decreases when increasing the radius of the alkali metal cation [102,104,105,106]. Moreover, the membrane conductivity is limited by ion transfer. The larger the cation radius, the more hindered the ionic transfer is [82,97,102,106]. This determines an increase in conductivity from the cesium to lithium forms of membranes. The reasons for the lower conductivity of the Ba^2+^ forms of membranes should be specially mentioned. A twice larger charge of Ba^2+^ ions determines the concentration of barium ions in the membrane, which is half as much as compared to the alkali metal cations. At the same relative humidity, a lower barium content in the membranes results in less water absorption by the membranes. In addition, the activation energy of ion transfer increases sharply when increasing the cation charge, which determines the higher strength of its bonds with the environment [82,100,102].

## 4. Ion Transfer in H^+^-Forms of Membranes

Membranes in H^+^-form (when the counterions are protons) are perhaps the most often discussed in the literature. For these, both solvation and ion transfer processes proceed somewhat differently than those for the salt forms of membranes. Therefore, in this review H^+^-forms of membranes will be discussed separately.

The main reason why protons deserve special attention as opposed to other cations is that they are elementary particles and their radii are extremely small. Protons do not have their own electrons and are located in the electron shell of electronegative atoms. In fact, the typical O–H distance is 1.0 Å, while the ionic radius of oxygen is 1.4 Å. In sulfonic acid ion-exchange membranes, protons usually bind to water molecules to form oxonium ions, H_3_O^+^. At the same time, these ions can also form strong hydrogen bonds with additional water molecules, resulting in the formation of more complex H^+^(H_2_O)_n_ ions. Among them, the most stable are H_5_O_2_^+^ and H_9_O_4_^+^ ions, which are formed stepwise during membrane hydration. Their formation is confirmed by ^1^H NMR data [72,107,108,109,110]. The authors of [111] reported a significant change in the properties of Nafion membranes with an increase in their hydration degree to above three water molecules per functional group. This number of water molecules was taken as the first hydration sphere. In this paper, to prepare anhydrous samples, Nafion membranes were dried at 105 °C. However, under these conditions, oxonium ions are usually retained in Nafion membranes—i.e., the hydration degree of three water molecules per functional group actually corresponds to the formation of H_9_O_4_^+^ ions. With a further increase in the water uptake, the mobility of proton-containing groups in these membranes increases significantly. Similar results were obtained by ^2^D T_1_ NMR relaxation measurements of the molecular motion of deuterated water in Aquivion E87-05, Nafion 117, and sulfonated-Radel proton-exchange membranes [112].

**Figure 3:**
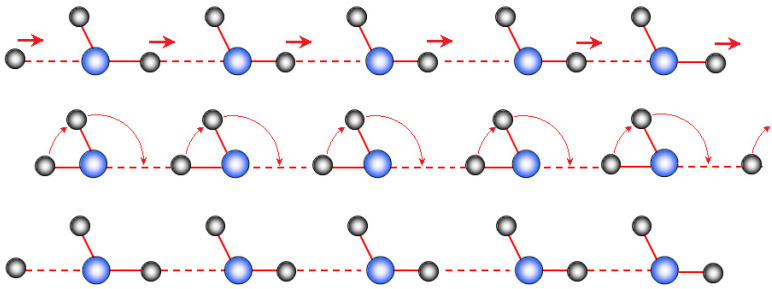
Scheme of proton transfer along the model chain of hydrogen bonds.

**Figure 4:**
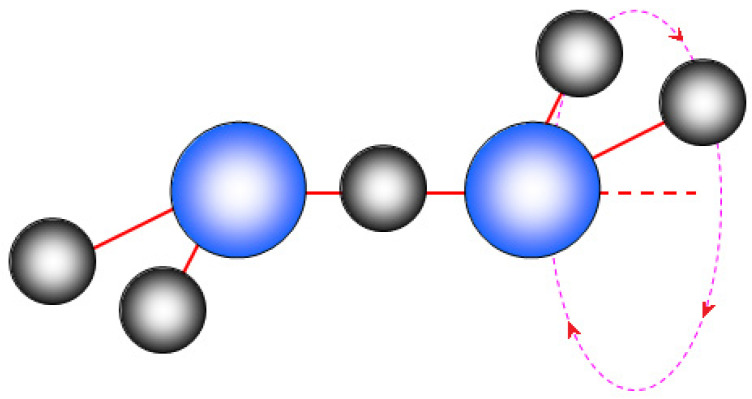
Scheme of the –OH_2_ fragment rotation of the H^+^(H_2_O)_n_ ion relative to the strongest hydrogen bond.

Hydrates of acids and acid salts are certainly the most common low-temperature solid proton-conducting electrolytes [113]. The proton moves in them in at least two stages, including the proton hopping along the hydrogen bond and the rotation of proton-containing groups. The scheme of proton transfer along the chain of hydrogen bonds is shown in Figure 3. The presence of two oxygen atoms on the hydrogen bond line (O–H…O) suggests two minima on the dependence of the potential energy on the proton position, corresponding to its localization at one of the oxygen atoms. The shorter the hydrogen bond, the lower the activation energy for proton hopping along this bond is. At the second stage, the rotation of the proton-containing group occurs (usually, this is the rotation of the oxonium ion or the –OH_2_ fragment of the H^+^(H_2_O)_n_ ion relative to the strongest hydrogen bond; Figure 4.). Moreover, during such rotation, the breaking of some and the formation of other H-bonds continuously takes place. Obviously, this process is facilitated when the strength of the formed bonds decreases. As shown in [114], the minimum activation energy of proton conductivity is achieved when the proton-acceptor ability of electronegative atoms participating in proton transfer is equal and the length of hydrogen bonds is of 2.78 Å. A hydrogen bond length close to this value is usually observed in ice-like structures. In this case, protons form defects, breaking the hydrogen bond network. Lone electron pairs of oxygen atoms in the H_5_O_2_^+^ ion cannot form hydrogen bonds with protons of other water molecules. This greatly facilitates their rotational mobility around the strongest hydrogen bond [114]. It is necessary to break only two hydrogen bonds for such a rotation. Its activation energy significantly reduces due to the gradual cleavage of some bonds and a gradual formation of other bonds since they are unsaturated and nondirectional.

Moreover, proton transfer is facilitated by the vibrational mobility of the hydrogen bond network. The proton hops at the moment of shortening of the H-bonds, while the rotation of proton-containing groups occurs at their lengthening. The so-called Grotthuss mechanism is based on cooperative effects [113,115]. Fast proton diffusion occurs in systems with a developed hydrogen bond network with intense vibrational mobility. The proton transfer is also facilitated by the change in the location of the short O–H…O bonds through the proton hops, followed by the formation of other short bonds due to the vibrations of the H-bond network. The Grotthuss mechanism explains the higher rate of proton transfer in ion-exchange membranes than that of other cations (Figure 2) [100,116,117,118,119]. This is also the reason for the violation of the regularity of the change in water diffusion coefficients with a radius of monovalent ion (Figure 1). In the case of H^+^-forms of ion-exchange membranes, not only diffusion of water is observed, but also diffusion of proton-containing groups of H^+^(H_2_O)_n_ ions with an anomalously high mobility of protons in aqueous solutions. As a result of the fast exchange between them, only one line of highly mobile proton-containing groups was observed in the ^1^H NMR spectra.

**Figure 5:**
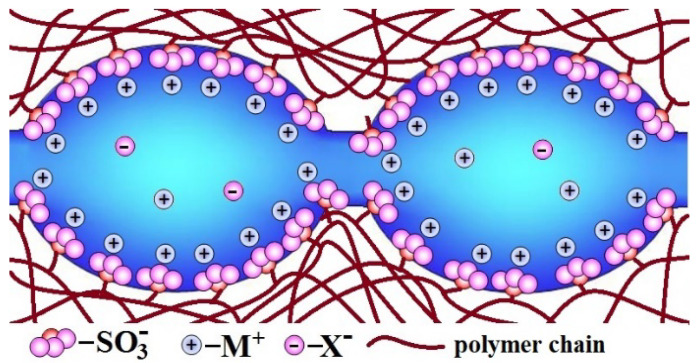
Structure of pores and channels in the ion-exchange membranes (adapted from [123]). Reproduced with permission from [123]. Springer, 2013.

At the same time, it should be noted that the “solution” in the pore and channel systems of ion-exchange membranes is not homogeneous. The pore walls of membranes have a negative charge due to the dissociation of SO_3_H groups. Due to the electrostatic interaction, most of the cations are localized near the pore walls within the Debye layer, which has a typical thickness of about 1 nm (Figure 5) [105,120]. On the contrary, in the central part of the pore, there is the so-called “electrically neutral solution” with a minimal concentration of counterions. Its concentration is close to that of the solution contacting with the membrane [121,122]. In particular, almost pure water was assumed to be in the pore center for the membrane contacting pure water.

Pure water freezes at 0 °C. However, this process is difficult to observe in membranes, due to the small radius of curvature and distortions of the ice-like structure by cations located near the pore walls. The authors of [124,125,126] concluded that water does not freeze at all in the pores of Nafion membranes. At subzero temperatures, water molecules can migrate to the membrane surface and crystallize on it. However, it is obvious that with an increase in the membrane water uptake and the pore size, the water should freeze near 0 °C. The data of low-temperature calorimetry for grafted membranes with a high degree of hydration show an endothermic transition with a maximum near 0 °C [127]. Based on the enthalpy of this peak, it was shown that only about half of the 44 water molecules per one functional group of the studied membranes melt near 0 °C. In this case, only an inflection on the temperature dependence of the ionic conductivity of these membranes was observed near 0 °C. This confirms that freezing water does not contain protons (charge carriers) [127], and proton transfer occurs in the Debye layer near the pore walls. At temperatures below this inflection, ice gradually crystallizes. Enrichment of the solution remaining inside the pores with cations leads to a decrease in the water freezing temperature. The gradual water freezing leads to a gradual increase in the activation energy of conductivity [127]. This inflection shifts to low temperatures for membranes with a hydration degree of <20 water molecules per functional group, to which most of the known membranes belong [127,128,129,130]. The activation energy of conductivity in the low-temperature range for these membranes is about 32 kJ/mol. However, in the high-temperature range, the activation energy increases to 28 kJ/mol with decreasing hydration degree to four water molecules per functional group [129], which hinders an inflection on the temperature dependences of conductivity. In [113,130,131,132] temperature-dependent ^1^H diffusion and spin-lattice relaxation NMR measurements were used to study water diffusion of Nafion membranes at different hydration degrees. The nonfreezing behavior of water molecules was shown at a hydration degree of <9 water molecules per functional group, while the freezing of free water molecules was observed at a higher hydration degree of ≥10 water molecules per functional group [113,132].

**Figure 6:**
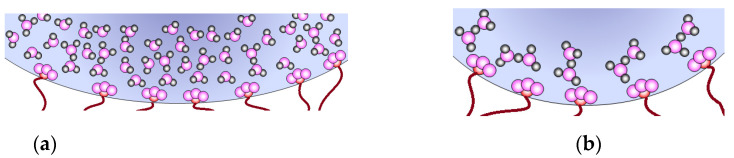
Scheme of proton transfer in membranes with high (**a**) and low (**b**) water uptake.

At high water uptake, –SO_3_H groups are completely dissociated and proton transfer occurs in an aqueous solution without the participation of functional groups (Figure 6a). At the same time, with water uptake decreasing, proton transfer becomes more and more difficult. H_5_O_2_^+^ ions cannot form hydrogen bonds with each other due to the low-proton-acceptor ability of their oxygen atoms and the distance between them increasing. All these factors lead to an activation energy that is too high for the direct proton transfer between them, and the –SO_3_^−^ groups of the membrane take part in the transfer (Figure 6b).

The Gierke model for ion clustering in perfluorinated sulfonic acid membranes described above was based on small-angle X-ray scattering studies [83]. The existence of narrow channels connecting the clusters (pores) was suggested as an explanation of the observed high ionic conductivity of Nafion membranes since the ionic pathways must obviously be present in some form to ensure long-range ion motion, which could not be realized for a system of isolated pores. This assumption was confirmed by comparing ionic conductivity and NMR spectroscopy data. The self-diffusion coefficients of lithium, sodium, and cesium cations in grafted membranes with high water uptakes, determined using PFG-NMR spectroscopy data, approach those of aqueous solutions [107]. The character of their change correlates well with that for the diffusion coefficients calculated from the conductivity using the Nernst–Einstein equation [107,129]. However, their values are more than an order of magnitude higher than those calculated from conductivity [107]. Obviously, in membranes most of the cations are in the pores. Thus, the NMR method provides information mainly about the cations located in them. Moreover, it is more sensitive to highly mobile ions characterized by a narrower NMR line width. Thus, the self-diffusion coefficients obtained from NMR data characterize ions in large pores. The lower diffusion coefficients calculated from the conductivity obviously characterize the mobility of cations moving in narrower channels connecting these pores [107]. These data unambiguously indicate that the ionic conductivity of membranes is limited by the transfer of ions in narrower channels connecting the pores.

The correctness of these conclusions is proved by the fact that the diffusion coefficients for aqueous solutions of alkali metal chlorides, determined using both methods, are equal and close to the values found by NMR for membranes samples [107]. It should also be mentioned that the type of ion-exchange membrane significantly affects the ratio of the diffusion coefficients measured using NMR spectroscopy and conductivity [133].

## 5. Selectivity of Transfer Processes in Ion-Exchange Membranes

**Figure 7:**
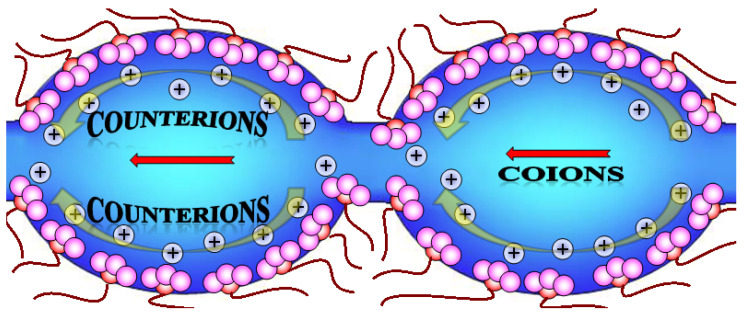
Scheme of ion transfer in the membrane pores and channel system.

As noted in the previous section, the ion distribution in membranes is not uniform, since counterions are predominantly localized in a thin Debye layer near the pore walls (Figure 7). They are selectively transferred in this layer. On the contrary, an electrically neutral solution located in the central part of the pores is equilibrated with the solution contacting with the membrane and can contain both counter- and coions in approximately equal concentrations [58,122]. It is this electrically neutral solution that ensures the nonspecific transfer of counterions and nonpolar or low-polarity molecules, which reduces the selectivity of transport processes [58].

With an increase in the water uptake, the size of membrane pores increases [58]. This also leads to an increase in the size of the channels, which limits the conductivity [113,134,135]. However, the water uptake especially increases due to an increase in the volume of the electrically neutral solution. The larger the volume of this solution, the higher the concentration of coions and low-polarity molecules can be in the membrane. In this regard, their flux is determined by the product of their concentration and their diffusion coefficient (Equation (1)) also increases. Therefore, with an increase in the water uptake, the proportion of nonspecific transfer also increases, which leads to a decrease in selectivity. To increase membrane conductivity, researchers usually simultaneously decrease membrane selectivity [136,137,138]. This is a universal law for different types of membranes and membrane processes. The main research task is to develop membranes [7], in which the optimal ratio of conductivity and selectivity for a given process is observed.

A recent study [139] reported the comparison of the transport properties of perfluorinated sulfonic acid membranes with both long and short side chains (Nafion and Aquivion type, respectively) with ion-exchange capacities ranging from 0.65 to 1.35 mEq/g. An increase in ion-exchange capacity leads to an increase in the water uptake and ionic conductivity [139]. Membrane conductivity measured in contact with water and at relative humidity of 32% increases by two and three orders of magnitude, respectively. The anion transport numbers simultaneously increase by approximately the same amount. At the same time, the gas permeability of membranes changes only several times [139]. This indicates that the transfer of gases to a large extent proceeds through the perfluorinated membrane matrix. Similar results were reported for gas transport through ion-exchange membranes with another type of polymer matrix [140].

Despite the outstanding combination of conductivity and selectivity of perfluorinated homogeneous ion-exchange membranes, much cheaper heterogeneous membranes are usually used in the most applications [141,142,143]. They are most often manufactured by rolling or hot-pressing ion-exchange materials (in particular, polystyrene sulfate) and a plastic binder. However, within such processes, macropores with sizes of about 1 μm remain between their granules [144,145,146,147,148,149]. These macropores, in addition to nanopores, lead to a lower selectivity of heterogeneous membranes [148,150,151]. An additional decrease in selectivity can often be determined by a reinforcing mesh, which is used to increase the strength of heterogeneous membranes [152].

In this regard, numerous attempts are being made to develop membranes with compositions similar to those of heterogeneous membranes, but without any macropores. For example, Neosepta® membranes are prepared by polymerizing styrene in the presence of polyvinyl chloride particles [153]. Another approach is related to the synthesis of graft copolymers, which is carried out by radical polymerization of styrene or other monomers inside a hydrophobic film activated by γ-rays, followed by sulfonation. This approach allows manufacturing of membranes with a wide range of ion-exchange capacities [154,155,156,157,158,159,160]. An approach using the softer UV-activation has also been proposed [161,162]. As a result, a number of membranes characterized by a combination of conductivity and selectivity close to those of the best commercial perfluorinated membranes were prepared [161,162].

As noted above, in recent years, much attention has been paid to the preparation of monovalent ion selective membranes. This is usually achieved by membrane coating with a number of layers with an alternating charge of functional groups [38,39,40,41,42]. The price for a multiple increase in selectivity is a simultaneous decrease in conductivity due to a large number of interfaces between the anion- and cation-exchange layers. As a result of salt bridge formation between their functional groups, the charge carrier concentration significantly reduces. At the same time, coating an anion-exchange membrane with a thin cation-exchange layer by chemical treatment of its surface allows one to achieve an increase in selectivity with a slight decrease in ionic conductivity [163,164,165].

## 6. Hybrid Membranes

To improve the transport properties of membrane materials, doping with inorganic nanoparticles is widely used. This approach is most often used to increase their conductivity or decrease the permeability of gases and/or methanol [166,167,168,169]. The synthesis of nanoparticles directly in membrane pores is the most effective. In this case, the formation of nanoparticles occurs simultaneously in almost all pores of membrane, while an increase in the dopant content leads to an increase in the particle size [170]. An improvement in conductivity is usually observed only at a low dopant content of about 2 vol% [171,172,173]. According to the model of limited elasticity of membrane pore walls, this is explained by the fact that the introduction of nanoparticles into membrane pores leads to an increase in their volume. At the same time, the channels connecting them and limiting the membrane conductivity also expand [174]. This model was developed based on the combination of NMR data and ionic conductivity of hybrid membranes [172]—e.g., conductivity of perfluorinated sulfonic acid MF-4SK membranes doped with a small amount of different oxides increases, while diffusion coefficients of proton-containing groups, determined by the PFG-NMR technique, increase in some cases, while others decrease compared to the pristine MF-4SK membrane. This emphasizes that the increase in conductivity cannot be attributed to the properties of the intraporeal solution, but to the expansion of the channels connecting the pores [107,172]. With an increase in the nanoparticle size, the elastic forces of the pore walls, caused by their deformation during expansion, increase according to the Hooke law, and the osmotic pressure becomes insufficient for their further expansion. As a result, the membrane water uptake decreases and regions that reduce the conductivity appear in the membrane pores. A mathematical description of a similar model was reported later [175]. 

As shown by NMR and DSC techniques, the Nafion/SiO_2_ and Nafion/Zr(HPO_4_)_2_ composite membranes exhibit a higher mobility of water molecules due to a higher hydration degree than dopant-free Nafion [176]. At the same time, using ^1^H NMR data the authors of [177,178] concluded that in the hybrid Nafion membranes with organosilica layered materials bearing different functional groups (–SO_3_^−^ and –PO_3_^−^) and the membranes based on polysulfone and layered doubled hydroxide there are two types of water: free water localized in the membrane pores and water sorbed by the dopant surface. 

**Figure 8:**
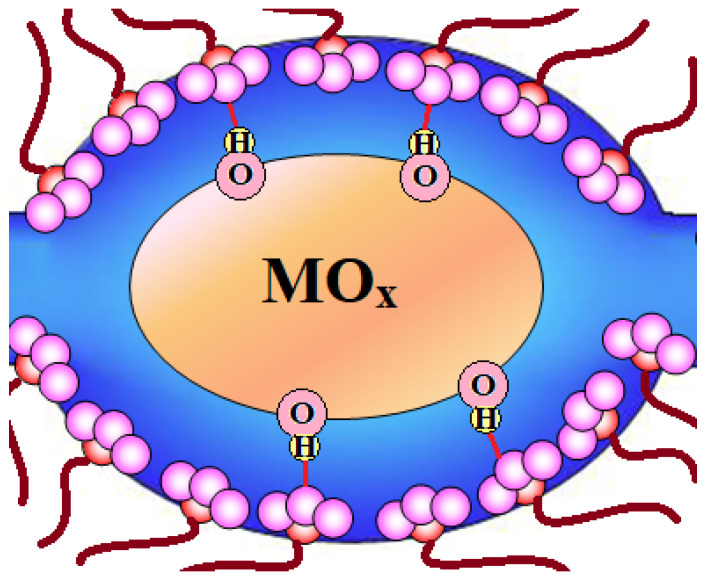
Scheme of the salt bridge (M^n+^–OSO_2_^−^) formation in hybrid membranes with base oxide nanoparticles.

The acidity of the dopant surface is essential too. A detailed study of ion-exchange membranes doped with nanoparticles of ZrO_2_, TiO_2_, and SiO_2_ oxides with increasing surface acidity showed that the ion-exchange capacity and conductivity of hybrid membranes increase in this series [179]. Basic oxides (ZrO_2_) reduce the ion-exchange capacity of the hybrid materials due to the formation of salt bridges (≡ZrO–H^+^–OSO_2_^−^), which exclude SO_3_^−^ groups from the ion exchange (Figure 8). At the same time, more acidic silica does not change it, since the dissociation of weakly acidic –Si–O–H groups is suppressed in the presence of a strong sulfonic acid (SO_3_H groups of the membrane). However, the ionic conductivity of the silica-doped membrane noticeably increases due to an increase in the water uptake and pore size [179]. At the same time, conductivity of the Na^+^-form of the membrane doped with ZrO_2_ is almost the same as for the pristine membrane. This is due to the destruction of salt bridges in an alkaline medium as a result of the proton removal from the membrane by the H^+^/Na^+^ ion-exchange and the hydrogen bond breaking. The selectivity of the studied hybrid membranes decreases in the same series. If, upon doping with silica, the apparent transport numbers of cations decrease by 7% relative to the pristine membrane, for the composite membrane with zirconia, they increase by 7% [179]. It is also possible to achieve an increase in the selectivity of the transfer processes by using other dopants exhibiting basic properties—e.g., polyaniline [180,181,182,183].

If the surface of the dopant particle contains acidic groups, an additional number of counterions (H^+^) are formed during their dissociation. This should contribute to an increase in both water uptake and conductivity. Moreover, the formation of the second Debye layer near the dopant particle surface with pronounced acidic properties can enhance the selectivity of transfer processes in hybrid membranes [170]. Typical examples of such dopants are heteropolyacids, their salts with alkali metal cations, or SiO_2_ particles with heteropolyacids [184,185,186,187,188]. Good results have also been obtained when sulfonated carbon nanotubes were used as dopants [189,190,191,192]. The presence of additional charge carriers and a decrease in the methanol permeability result in an increased power of direct methanol fuel cells based on such membranes [192,193,194]. The authors of [195,196] reported a fivefold increase in conductivity under reduced relative humidity (RH=20–25%) and a significant increase in the fuel cell power based on sulfonated graphene/Nafion membranes. Such trends are inherent in a number of hybrid membranes, and therefore they are often used in direct methanol fuel cells [176,197,198,199,200,201]. Moreover, a positive effect in fuel cell performance was reported for the composite membranes with carbon nanotubes containing basic imidazole groups on their surfaces [202,203].

## 7. Conclusions

Ionic and molecular mobility determine the basic trends of transfer processes which underlie membrane technologies widely demanded by modern industry. The transfer processes occur in the system of pores and channels of ion-exchange membranes, which are formed in them as a result of the self-organization and the sorption of solvents, especially water molecules. In this case, it is the solvation and the solvent content that determine both the rate of transfer and its selectivity.

The main method for studying transport processes in ion-exchange membranes is undoubtedly impedance spectroscopy. However, it characterizes only the overall ionic conductivity of membranes. These data are insufficient to describe the mechanisms of ionic and molecular transport in membranes. The use of NMR spectroscopy, including multinuclear techniques, relaxation data and pulsed field gradient NMR allows one to characterize the local environment of ions and molecules, their movements relative to each other, and rotational and translational mobility in different systems. The combination of these methods makes it possible to describe the mechanism of ionic and molecular mobility in a number of systems. This knowledge, in turn, should form the basis for a more thorough understanding and improvement of approaches to the synthesis of membrane materials.

At the same time, NMR spectroscopy, which has found wide application in chemistry, is often used only to prove the structure of substances and its possibilities are not fully used—e.g., NMR spectroscopy is relatively rarely used to study the processes of solvation and transfer in membrane materials, although as was shown in this review, it provides important information, including an explanation of changes in the transport properties of ion-exchange membranes. Therefore, it can be assumed that the wider use of NMR spectroscopy for the study of membranes will contribute to the progress in the area of membrane processes and technologies.

## Data Availability

Not applicable.
